# Assessment of the accuracy of portable monitors for halitosis evaluation in subjects without malodor complaint. Are they reliable for clinical practice?

**DOI:** 10.1590/1678-7757-2016-0305

**Published:** 2017

**Authors:** Denise Pinheiro Falcão, Priscila Carvalho Miranda, Tayana Filgueira Galdino Almeida, Monique Gomes da Silva Scalco, Felipe Fregni, Rivadávio Fernandes Batista de Amorim

**Affiliations:** 1 Universidade de Brasília, Faculdade de Medicina, Programa de Pós-Graduação em Ciências Médicas, Brasília, DF, Brasil; 2 Serviço Público de Saúde do Distrito Federal, Brasília, DF, Brasil; 3 Harvard Medical School, Laboratory of Neuromodulation & Center for Clinical Research Learning, Charlestown, Massachusetts, United States

**Keywords:** Halitosis, Diagnosis

## Abstract

Halitosis is defined as a foul odor emanated from the oral cavity, with great impact in quality of life and social restraints. Recently, the use of Breath Alert™ in research increased significantly. Halimeter™, another portable device, is often used in clinical practice. Nevertheless, not many studies have verified the accuracy and compared the results of both devices simultaneously. Objective: To verify the accuracy of Breath Alert™ and Halimeter™ in patients without chief complaint of halitosis, using the organoleptic test (OT) as "gold standard." The second aim was to verify whether their concomitant use could enhance the diagnostic accuracy of halitosis. Material and Methods: A cross-sectional analytical study was performed. The quality of expired air of 34 subjects without chief complaint of halitosis was assessed. Two experienced examiners carried out the OT. Afterward, a third blinded examiner performed Halimeter™ (HT) and Breath Alert™ (BA) tests. Results: The OT identified halitosis in 21 subjects (62%). The area under the ROC curve (95% confidence interval) was 0.67 (0.48-0.85) and 0.54 (0.34-0.75) for HT and BA, respectively. The accuracy for HT and BA was 59% and 47%, respectively. The combined usage of HT and BA provided 11 positive results, being 9 subjects (43%) out of the total of 21 positive cases. Conclusions: Halimeter™ and Breath Alert™ were not able to diagnose halitosis in non-complainer subjects at the same level as the organoleptic examination, since their accuracy were low. Our results suggest that such portable devices are not reliable tools to assess halitosis and may neglect or misdiagnose a considerable number of patients in clinical practice.

## Introduction

Halitosis is a universally experienced condition that has a variety of physiologic, pathologic and adaptive etiologic factors and affects nearly 15 to 30% of the population^5^ ^,^ ^7^ ^,^ ^11^ ^,^ ^21^. The gold standard method to evaluate halitosis is the organoleptic or "sniff" test (OT), which access all oral odorants collectively. In this test, the examiner uses the sense of smell to detect malodor and subjectively score patients’ halitosis^21^. It has some important drawbacks, such as being dependent on someone's interpretation about the quality of the odor and the offensiveness score of the smell. Subjective measures are an issue in research, since objective data are more likely to be standardized. In addition, the organoleptic test requires calibration and can be an embarrassing procedure^1^ ^,^ ^15^. Therefore, it is critical to access the sensitivity and specificity of other methods in order to decrease the need of performing OT.

Many efforts have been made to create reliable and objective methods to evaluate halitosis. Halimeter™ (Interscan Corporation, Chatsworth, CA, USA) is a portable monitor that measure the amount of sulphur compounds responsible for bad breath. However, it still does not meet all the requirements to be considered an "ideal" device in the assessment of halitosis^25^. Breath Alert™ is another device that (Tanita Corporation, Tokyo, Kantõ, Japan) has been gaining special attention in clinical practice^3^ ^,^ ^6^ ^,^ ^12^ ^,^ ^13^ ^,^ ^17^. It is a small handheld breath-checking equipment that measures and calculates the volatile sulphur compounds and hydrocarbon gases in expired air. Nevertheless, not many studies have verified its accuracy and relevance in clinical practice.

We believe not enough studies have accessed the breath odor quality in patients without chief complaint of halitosis. Since some patients with halitosis might be unaware of their condition, we aimed to study a population more representative of daily clinical practice. Thus, the main purpose of this study was to access simultaneously the accuracy of Breath Alert™ and Halimeter™ in patients without chief complaint of halitosis, using the OT as "gold standard." We also hypothesized that the concomitant use of both methods could enhance the diagnostic accuracy of halitosis, and thus could be helpful in decreasing the use of OT in clinical practice.

## Material and methods

The study design has followed the guidelines for quality of evidence for studies of diagnostic accuracy^19^. A cross-sectional analytical study was performed using a convenience and consecutive sample. The human subject protocol was in accordance with the principles laid down in the Declaration of Helsinki and was approved by the correspondent Institutional Board (033/04). Informed consent was obtained from those who accepted the invitation. Participants were examined in two moments. A periodontist performed intraoral examination and instructed about the following visit for halitosis assessment. In the second visit, two experienced examiners who have been working with halitosis for more than 15 years carried out the OT. Considering that the unit of measurement is on a categorical scale, reliability needed to be properly assessed as a measure of agreement. The examiners were calibrated until the level of concordance reached 80%. Hence, the kappa index was 0.8 (great agreement level). Both examiners were blinded regarding the oral condition of the patient. Afterwards, another examiner performed Halimeter™ (HT) and Breath Alert™ (BA) tests in a blind fashion (oral status and OT results).

### Subjects

The sample was composed of men and women who visited the private office of one of the authors consecutively during one month. Subjects who fulfilled the following criteria were eligible: not diagnosed with gingival and or chronic periodontal diseases based on the American Academy of Periodontal Classification^2^ and without complaint of halitosis. In addition, oral-pharynx inflammatory processes, cold or flu, oral fistula and current use of antibiotics treatment (within the previous 3 weeks) represented the exclusion criteria.

The subjects were informed about the breath malodor examination and received written instructions for the next appointment. They were instructed to not consume garlic, onion, spicy foods, and alcohol. Gargling oral rinse and breath fresheners during 24 hours before the breath examination were not allowed as well. They were also instructed to not smoke during the 12 hours preceding the evaluation. Within the previous 2 hours, the subjects had to abstain the use of aromatic beverage, such as tea or coffee. They were told to have a meal and to perform their usual oral hygiene practices 2 hours before the assessment. At the day of halitosis assessment, the use of any scented cosmetic, such as perfume and aftershave, was not allowed once it could interfere in the examiners’ olfactory sense and with the equipment sensors.

### Organoleptic test

Examiners were instructed to postpone the assessment in the presence of any unpredictable situations that could lead to olfactory disturbance, such as postnasal drip, rhinitis, sinusitis or cold. Volunteers were required to close their mouth and refrain from talking for 3 minutes before the evaluation. Aiming to standardize the assessment distance, the edge of a 15 cm length rule was placed on the mentolabial sulcus of the patient and the other edge bellow the nostrils of the examiner. Subjects were instructed to slowly exhale their breath by saying "raaaaaaaaaus" until they could feel their lungs empty.

### Volatile sulphur compound (VSC) measurement

Measurements of total VSC of expired air were taken with a portable industrial sulphide monitor Halimeter™ (Interscan model 1170). For this purpose, the instrument was zeroed on environment air and patients were instructed to keep their mouth closed, as previously required for the OT. The breath sample was assessed three times, as recommended by the manufacturer. A disposable straw connected to the device sensor was inserted into the patient's mouth in a standardized distance of 4 centimeters. The patient was instructed to close his mouth with the straw inside by keeping the lips on a leaked silicone cylinder. The results ≥80 parts-*per*-billion (ppb) were considered positive for HT.

### Breath Alert™ assessment

Before each measurement, Breath Alert™ was gently waved in the air 4-5 times to remove any odors or moisture left in the device, as recommended by the manufacturer. Patients received the same instructions regarding keeping their mouth closed for three minutes. Afterwards, the device was turned on and the patients’ thumb was positioned on the front of the unit to help directing the sensor toward the mouth. As soon as the display showed the word "start," patients expired the air, as recommended in the OT. This study considered the BA scores 1 and 2 as negative, and scores 3 and 4 as positive for halitosis.

### Statistical analysis

Data analyses were carried out using Statistical Package for Social Sciences SPSS™ version 20.0 for Windows™ (SPSS Inc./IBM Group, Chicago, IL, USA). Chi-squared test with a significance level of 5% verified the difference among OT, HT and BA tests. In order to access and compare the diagnostic accuracy of these three tests, receiver-operating characteristic (ROC) was carried out, and the OT was considered the “gold standard.”

## Results

The sample was composed of 34 adult patients (44% men and 56% women), mean age of 44.2±14.6 years (range: 25 to 75) with mean value parts-per-billion of volatile sulphur compounds of 52 (range: 1 to 167).

**Figure 1:**
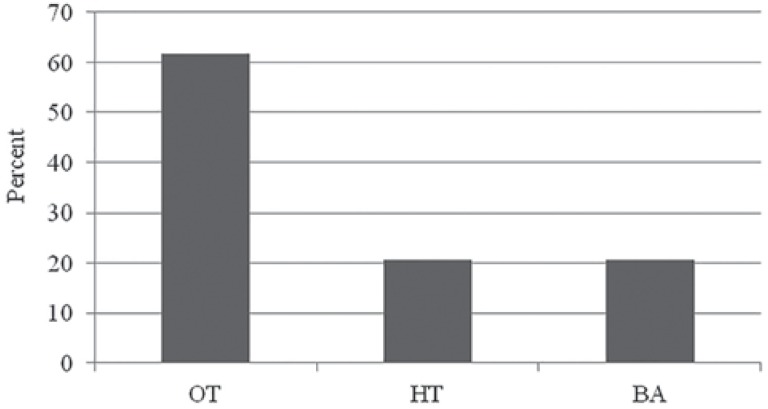
Prevalence of halitosis positive diagnosis performed by the Organoleptic Test (OT), Halimeter™ (HT) and Breath Alert™ (BA) tests (p≤.001)

The OT was positive for 21 participants (62%) (Figure 1). The area under the ROC curve was 0.67 (95% confidence interval, 0.48 to 0.85) and 0.54 (95% confidence interval, 0.34 to 0.75) for HT and BA, respectively. The sensitivity was 33% and 24% for HT and BA, respectively (Table 1).

**Table 1:** Diagnostic results of Breath Alert™ and Halimeter™ tests

	Halimeter™	Breath Alert™
Sensitivity (%)	33	24
Specificity (%)	100	85
Accuracy	59	47
PPV^a^	100	71
NPV^b^	48	41
PLR^c^	0.33	1.44
AUC (95% CI)^d^	0.67 (0.48 to 0.85)	0.54 (0.34 to 0.74)

a PPV: positive predictive value;

b NPV: negative predictive value;

c PLR: Positive likelihood ratios;

d AUC (95% Cl): area under the curve (95% confidence interval)

**Figure 2:**
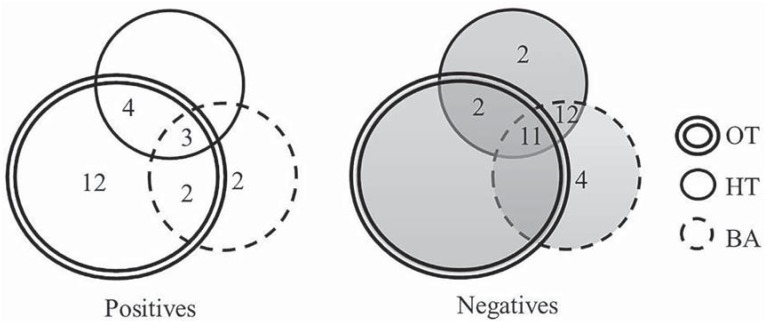
Positive and negative absolute frequency for Halimeter™ (HT) and Breath Alert™ (BA) in relation to Organoleptic Test (OT)

The combined usage of HT and BA provided 11 positive results, being 9 (43%) out of the total of 21 positive cases. Using simultaneously all the methods (OT, HT, BA), three subjects were identified as positive and eleven as negative in the same way. (Figure 2). The ppb mean value of VSC of the three positive cases was 167, 156 and 110 ppb (data not shown).

## Discussion

This study aimed to access and compare the accuracy/sensitivity of two portable devices and OT in non-halitosis complainer subjects. Furthermore, it was verified whether the combined methods used could enhance accuracy for clinical diagnosis of halitosis. The sample was composed of halitosis’ non-complainers since those who are unaware of their condition can have halitosis related to systemic diseases. In addition, halitosis affects individuals’ social relations significantly. Therefore, this condition of utmost importance requires special attention. Our results demonstrated that 62% out of the 34 patients presented bad breath with any complaint or when inquired about odor breath quality.

Body odor elicits a great concern including oral odor. Hygiene and beauty products sales increased 12% between 2002 and 2003 in Latin America. A significant increase in the *per capita* consumption of toothpaste, toothbrush, mouth rinse and dental floss has been estimated from 1992 to 2002, respectively at rates of 38.3%, 138.3%, 618.8% and 177.2%^10^. In this context, the general population can easily purchase some inexpensive palm-sized monitors, such as Breath Alert™, to quickly access odor breath.

The levels of compounds in exhaled mouth air depend on the type of microflora and the available metabolic substrates in the mouth. VSC has been considered the main contributor to oral malodor due to their low odor detection thresholds and high odor power. However, many other lower odor power organic compounds are present in the expired air, such as ketones, fatty acids, amines, alcohols, aldehydes and hydrocarbons

In some cases, the threshold levels are attained by only one of the portable devices. In such cases, malodor can also be organoleptically detected. Thus, the sulphur monitors can indicate that there is no objectionable malodor in case of pseudo-halitosis or halitophobia. It is well established that OT is the "golden method," but it must be mentioned that oganoleptic scores are often regarded as subjective, especially by patients with an uncertain diagnosis.

Even though HT and BA measure only a specific odor subgroup out of all odorant groups related to malodor, it could be hypothesized that the assessment of mouth odor with both devices simultaneously could increase the accuracy of portable monitors. Unfortunately, our results were not able to indicate any advantage to reach a final diagnosis by using both devices. The results showed the specificity of the tests was high (100% and 85% for HT and BA, respectively), and the sensitivity was too low (33% and 24% for HT and BA, respectively) for allowing an examiner to substitute the OT for either the single or the combined use of HT and BA (Table 1), though HT and BA together identified 11 out of 21 positive cases (Figure 2).

Measurement validity is a critical factor of evidence-based practice to assure that our assessment tools provide us reliable information for decision-making. Diagnostic test should be executed only if its result can change treatment decisions^22^. In this study, the accuracy of HT and BA were both low (Table 1), which showed that none of them could be reliable for the diagnosis of halitosis in the studied sample. When a diagnostic tool has high sensitivity, its negative test result will indicate the absence of the abnormal condition. On the other hand, when specificity is high and the test result is positive, the probability of an abnormal condition will be strong.

The prevalence of positive halitosis diagnosis through the OT was much higher (62%) than HT or BA (21%) (Figure 1). From those cases diagnosed with halitosis by the OT, HT identified only 7 (33%) out of 21 cases, while BA correctly diagnosed 5 (24%) positive cases (Figure 2). The mean VSC value found for such seven cases with halitosis diagnosed through HT alone were 137 ppb, being the minimum value 94 ppb and the maximum one 167 ppb (data not shown). From the total of HT positive results, only three cases were in accordance with both OT and BA (Figure 2), and the ppb value for their VSC were 167, 156 and 110 ppb (data not shown).

VSC have the potential role of promoting halitosis, though other volatile compounds can also compromise the odor quality of expired air, such as volatile short-chain fatty acids, polyamines, alcohols, phenyl compounds, alkanes, ketones and nitrogen compounds^20^ ^,^ ^21^. However, little attention has been given for the later above-mentioned volatile compounds due to their low volatility. Thus, it is believed that those compounds have little influence in impairing the quality of the expired breath. Although in this study patients did not report any complaint regarding their breath odor, and that only seven patients out of the total of 34 subjects (20.5%) presented VSC values greater than the cut off value (80 ppb) established by the manufacturer, the offensive smell was present in another more 14 patients. Thus, 66% of the halitosis diagnosed patients in this sample did not present VSC as the main responsible for the bad breath. In this way, more attention should be paid in other volatile compounds as mentioned above.

Regarding the sensitivity and specificity values of both methods, HT and BA (Figure 1), the first one showed better performance, as reflected in its accuracy value (59%). However, not even the HT was reliable enough to be used alone. This device does not detect several volatile compounds nor some VSC found in halitosis, as previously discussed^23^, while the OT is able to perceive all of them. Differences regarding the presence of halitosis measured by organoleptic test, HT and BA have been also reported in recent studies^13^, but usually there is great positive correlation between organoleptic scores and VSC values^22^.

OT is considered the gold standard for the clinical diagnosis of halitosis^5^ since it reflects human perception, and, to date, only humans can judge the acceptability of the mixed odors emanating from the mouth^8^. However, it has already been mentioned that OT can transmit diseases to the operator through the expelled air^14^. In addition, the OT method can constrain both the patient and the judge once it requires smelling others’ exhaled breath. Additionally, OT has been criticized for its subjectivity level even after getting good results in olfactory capability test, and after a rigorous calibration of examiners^6^ ^,^ ^24^. It also might be affected by environmental conditions. Besides those issues, the examiner must postpone the examination in case of any sign that could promote their olfactory disturbance, such as post-nasal drip, rhinitis, sinusitis or cold. Since there is a preparation for halitosis assessment, the need of postpone might cost and be time-consuming for both patient and examiner. Despite all the drawbacks, organoleptic measurements are still the gold standard for assessing halitosis^13^.

Another potential use and development of these tests is to detect subclinical halitosis when offensive odors are not detected by examiner olfaction regardless of patient's complaint. In these cases, there is already a low amount of VSC or even other volatile compounds perceived by the retronasal sense of smell of the patient that additionally may lead to clinical halitosis in the future. In this context, new studies should be conducted for the establishment of the amount of VSC able to cause what has been considered as subclinical halitosis. In addition, these devices could also be used to differentiate types of halitosis related to great amount of VSC *versus* other volatile compounds perceived in OT for clinical halitosis cases. These might help to delineate treatments in the future.

Although the study sample was relatively small and results were straightforward and limited, they do indicate potential limitations of both commercially available devices tested. During clinical practice, the most useful tool for interpreting diagnostic tests is the likelihood ratios, and values close to 1 indicate that the test does not provide much information^9^. Thus, supposing HT would present high positive likelihood ratio, while BA would have high negative likelihood ratio or *vice-versa,* we could assume that their associated usage would allow diagnosing halitosis effectively. However, this study showed the combined use of HT and BA was not a reliable tool for halitosis diagnosis. Even though HT in conjunction with the organoleptic test has been considered an effective method for diagnosing oral malodor^4^, our results did not support this finding.

Although the costs of these devices are feasible in relation to expenditure, they do not provide sufficient valid information to be used in a diagnostic context. Thus, our data are in accordance with other studies that have considered the organoleptic test to be the "gold standard" clinical method for detecting the presence or absence of halitosis^7^ ^,^ ^16^ ^,^ ^18^ ^,^ ^25^. The need to develop a reliable clinical method that can be used as a substitute for the organoleptic test for halitosis detection remains.

## Conclusion

Considering the design and drawbacks of this study, we conclude that the measurement of the VSC levels detected by portable devices can be used as an adjuvant tool with OT in subjects without malodor complaint. In spite of the great improvement of such devices in the last years, OT remains the "gold standard" method for the diagnosis of bad breath. It is known that Halimeter™ and Breath Alert™ have great specificity, nonetheless, their accuracy have not reached the desired confidence for clinical practice yet. Hence, they are not reliable methods to diagnose halitosis in non-complainer patients at the same level as the organoleptic examination. In addition, they cannot be considered reliable even when their results are gathered and analyzed together. Even though the use of the portable devices can lead to a considerable number of negative results, their value must be highlighted as an important tool for malodor screening. Finally, these devices can be also very useful for patient's follow-up and to differentiate distinct types of halitosis, such as those caused by great amount of sulphur compounds from others (i.e. organic volatile compounds).
